# Variation in *NOD2* Augments Th2- and Th17 Responses to Myelin Basic Protein in Multiple Sclerosis

**DOI:** 10.1371/journal.pone.0020253

**Published:** 2011-05-20

**Authors:** Chris Juul Hedegaard, Christian Enevold, Finn Sellebjerg, Klaus Bendtzen, Claus Henrik Nielsen

**Affiliations:** 1 Institute for Inflammation Research, Department of Rheumatology, Copenhagen University Hospital, Rigshospitalet, Denmark; 2 Danish MS Research Centre, Department of Neurology, Copenhagen University Hospital, Rigshospitalet, Denmark; Universita di Sassari, Italy

## Abstract

Variations in the gene for the nucleotide-binding oligomerisation domain (NOD) 2 have been associated with Crohn's disease but not multiple sclerosis (MS). Here we investigate the effect of three polymorphisms in the *NOD2* gene (rs5743277, rs2066842 and rs5743291) on cytokine production and CD4+ T cell proliferation elicited by human myelin basic protein (MBP) in blood mononuclear cell (MNC) cultures from 29 patients with MS. No polymorphism was observed at rs5743277. No associations with the rs2066842 polymorphism were found. Concerning rs5743291, none were homozygous for the minor allele. Seven of 29 (24%) patients were heterozygous, and five of these (71%) exhibited increased MBP-induced CD4+ T cell proliferation versus four of 22 (18%), who were homozygous for the major allele (p<0.04). Interleukin (IL)-5 was induced by MBP in MNC from the same five carriers versus two (9%) homozygotes (p<0.004); four carriers (57%) versus three non-carriers (14%) exhibited IL-17 responses to MBP (p<0.04). By contrast, we found no association between the polymorphisms investigated and interferon-gamma-, tumor necrosis factor-alpha-, IL-2, -4- or IL-10 responses to MBP. These results indicate that the rs5743291 polymorphism influences T helper (Th) cell 2- and Th17 cell responses in MNC from MS patients.

## Introduction

Pattern recognition receptors (PRRs) recognizing “non-host” molecular patterns play an essential role in recognition of microbial pathogens by cells of the innate immune system [1]. The nucleotide-binding oligomerization domain (NOD)-like receptor NOD2 is an intracellular PRR recognizing the bacterial peptidoglycan derivative muramyl dipeptide (MDP), reviewed in Mathews et. al. [2]. NOD2 comprises two N-terminal caspase recruitment domains (CARD), a central nucleotide binding domain, referred to as NACHT, and a C-terminal leucine-rich repeat (LRR) domain [3]. The effector of NOD2 is the LRR domain, which upon activation by MDP induces production of proinflammatory cytokines by signaling through nuclear factor kappa B (NF-kappaB), reviewed in Takeuchi et. al. [4].

Mutations in NOD2 gene confer susceptibility to several chronic inflammatory disorders, including Crohn's disease and Blau syndrome [5], [6], [7]. Thirty non-conservative mutations associated with Crohn's disease have been identified within the *NOD2* gene, but only three are common, namely those leading to the Arg702Trp and Gly908Arg substitutions, and a frame shift mutation (3020insC→1007fs) resulting in a truncated version of NOD2. These three variants are all situated in the LRR domain of NOD2, mutations in which result in failure to activate the NF-kappaB signal transduction pathway upon binding of NOD2 ligands [3], [5], [6], [7], [8]. The 3020insC mutation lowers interleukin (IL)-10 syntheses by inhibiting the activity of heterogeneous nuclear ribonucleoprotein A1 [9], [10], [11]. Mutations in the NACHT domain, on the other hand, result in elevated MDP-induced up-regulation of NF-kappaB [12]. The three mutations in the LRR mentioned above do not appear to confer susceptibility to multiple sclerosis (MS) [13].

Stimulation of human dendritic cells with MDP has been shown to enhance NOD2-mediated production of IL-23, IL-1 by the dendritic cells and, in turn, promote IL-17 production by memory Th17 cells [14]. IL-17 has been shown to be involved in chronic inflammatory diseases, including MS and Crohn's disease [15], [16], [17], [18], [19], [20], [21].

We recently demonstrated a close association between active cerebral lesions in multiple sclerosis (MS) and CD4+ T cell proliferation and mononuclear cell (MNC) production of IL-17 and IL-5 induced by myelin basic protein (MBP) ex vivo [18]. Proliferative CD4+ T cell responses and production of IL-17, IFN-γ, and IL-5 and IL-4 were only seen in a subgroup of high-responder patients and not in healthy controls. In view of the reported influence of NOD2-signaling on IL-17 production, we have here examined whether certain single nucleotide polymorphisms (SNPs) in the *NOD2* gene were characteristic for high-responder patients, and demonstrate that a G>A substitution in exon 9 of the *NOD2* gene is associated with MBP-induced CD4+ T cell proliferation, IL-5 production, and IL-17 production, but not with increased Th1 -responses.

## Materials and Methods

### Ethics Statements

The study was approved by the Scientific Ethics Committée of Copenhagen and Frederiksberg, and prior to blood draw, all participating subjects gave their verbal and written informed consent.

### Subjects

The study included 29 patients with MS, 20 women and nine men, median age: 34 yrs, range: 23-44 yrs (Table 1), and 27 of these patients were diagnosed with relapsing-remitting MS (RRMS) and two with clinically isolated MS syndrome. All patients were untreated at the time of inclusion. None had been treated with immunosuppressive drugs, and none had been treated with glucocorticoids within four weeks of study entry.

**Table 1:** Patient characteristics.

Patient #	Gender	Age	Diagnosis^1^	MBP-induced CD4+ T cell proliferation^2^	NOD2 polymorphisms		
					rs2066842	rs5743277	rs5743291
1	M	23	RRMS	-	C/C	C/C	G/G
2	M	24	RRMS	-	T/T	C/C	G/G
3	F	25	RRMS	+	C/**T**	C/C	G/**A**
4	F	35	RRMS	+	C/C	C/C	G/G
5	F	25	CIS	-	C/**T**	C/C	G/G
6	F	28	RRMS	+	C/C	C/C	G/**A**
7	F	35	RRMS	+	C/**T**	C/C	G/**A**
8	F	28	RRMS	-	C/**T**	C/C	G/**A**
9	M	29	RRMS	+	C/**T**	C/C	G/A
10	F	29	RRMS	+	C/C	C/C	G/G
11	M	30	RRMS	-	C/**T**	C/C	G/G
12	F	29	RRMS	-	C/C	C/C	G/G
13	M	32	RRMS	-	C/C	C/C	G/G
14	F	33	RRMS	-	C/**T**	C/C	G/G
15	M	35	RRMS	-	C/**T**	C/C	G/G
16	F	34	RRMS	-	C/**T**	C/C	G/G
17	F	36	RRMS	-	C/C	C/C	G/G
18	F	37	RRMS	-	C/**T**	C/C	G/G
19	F	38	RRMS	+	T/T	C/C	G/G
20	F	38	RRMS	-	C/C	C/C	G/G
21	M	39	RRMS	-	C/C	C/C	G/G
22	F	30	RRMS	+	C/**T**	C/C	G/G
23	M	39	RRMS	-	C/**T**	C/C	G/G
24	F	39	CIS	-	C/C	C/C	G/**A**
25	M	41	RRMS	-	C/**T**	C/C	G/G
26	F	27	RRMS	+	C/C	C/C	G/**A**
27	F	42	RRMS	-	C/C	C/C	G/G
28	F	42	RRMS	-	C/C	C/C	G/G
29	F	44	RRMS	-	C/**T**	C/C	G/G

Minor alleles are marked in bold.

1 RRMS: relapsing-remitting MS, CIS: clinically isolated syndrome.

2 Positivity was defined as more than 1% dividing CD4+ T cells after allowance for background proliferation.

### Cells and serum

Blood was collected in lithium-heparin tubes and dry Vacutainer tubes (BD Bioscience, Brøndby, Denmark). MNCs were isolated from the former by density centrifugation on Ficoll-Hypaque, Lymphoprep® (Nycomed, Oslo, Norway), and serum was isolated from the latter tubes. The MNCs were labeled with 2 µM 5,6-carboxyfluorescein-diacetate-succinimidyl-ester (CFSE; Molecular Probes/Invitrogen, Taastrup, Denmark) and cultured in 96-well Nunclon™ flat-bottomed MicroWell^TM^ plates (Thermo Fischer Scientific, Slangerup, Denmark).

### Stimulation of MNCs with MBP

Cultures of the labeled MNCs (5×10^5^ cells per well) were grown in RPMI 1640 (Biological Industries, Kibbutz Beit Haemek, Israel), containing 50 µg/mL Gentamycin (Gibco/Invitrogen), 2 mM glutamine (Gibco/Invitrogen), and 30% (v/v) serum to a final volume of 150 µL per well. The cells were grown for 10 days in the absence of antigen (negative control), or in the presence of purified human MBP (HyTest, Turku, Finland) at a concentration of 30 µg/mL. Culture supernatant, 85 µL, was removed on days one and seven for assessment of cytokine contents, and the cultures were supplemented with 100 µL of RPMI at these time points.

The MBP preparation used was tested by mass spectrometry and found to be >95% pure, and the major contaminant being hemoglobin. No traces of the NOD2 ligand MDP were found, and all the measured MBP-induced responses were abrogated by boiling the MBP preparation showing that the responses observed were not induced by contaminating LPS [22].

### Measurement of cytokines

The content of the cytokines IL-4, IL-5, IL-10, IL-2, tumor-necrosis factor (TNF)-alpha and interferon (IFN)-gamma in the culture supernatants and patient sera were measured by flow cytometry using the cytometric bead array (CBA) Th1/Th2 kit and the corresponding software (CellQuest, BD Bioscience) according to the manufacturer's protocol, except that three-fold dilutions of all reagents were used. IL-17 was measured with an IL-17 singleplex kit (Invitrogen) using a Luminex 100 IS (Luminex corp., Austin, Texas, USA). Data analyses were carried out with StarStation version 2.0 software (Applied Cytometry Systems, Sheffield, UK).

A positive cytokine response was defined as ≥5 pg/ml after subtraction of the production in the absence of stimulating antigen.

### Measurement of proliferation

Measurement of CD4+ T cell proliferation was carried out as in Nielsen et. al. [23] In brief, cell divisions were tracked on the basis of the CFSE-content, which is halved upon each cell division. Cells with a fluorescence intensity of less than one half of the intensity of undivided cells were considered proliferating cells. T helper (Th) cells were identified as cells within a morphological (forward-/side scatter) lymphocyte gate staining with PerCP-anti-CD4 antibodies (BD Bioscience). Proliferation was measured at day 10 using a FACScalibur flow cytometer (BD Bioscience). Responders to MBP were defined as individuals with ≥1% divided CD4+ T cells after allowance for the background proliferation occurring in the absence of stimulating antigen.

### Genotyping

Genotyping was performed using in-house bead-based multiplex single nucleotide polymorphism (SNP) assays as described elsewhere [24]. Briefly, allele-specific primers were labeled in an allele-specific primer extension (ASPE) reaction, using polymerase chain reaction-amplified SNP-sites as their target sequences. The labeled ASPE-primers were subsequently hybridized to MicroPlex-xTAG beadsets for detection and counting on the Luminex 100 IS platform. Three SNPs were investigated in NOD2: rs2066842, rs5743277, and rs5743291 (Table 2).

**Table 2:** Influence of the rs5743291 polymorphism on MBP-induced CD4+ T cell responses.

rs5743291	G/G^1^(n = 22)		G/A(n = 7)		Test results^2^
Response to MBP	Yes	No	Yes	No	G/G vs. G/A
CD4+ T cell proliferation^3^	4	18	5	2	P<0.02
IFN-gamma production^4^	9	13	5	2	P<0.22
IL-2 production^4^	5	17	3	4	P<0.36
IL-4 production^5^	5	17	3	4	P<0.36
IL-5 production^4^	2	20	5	2	P<0.004
IL-17 production^4^	3	19	4	3	P<0.04

TNF-alpha was produced in all cases and IL-10 in 28 out of 29 cases. These cytokines were not included in the table.

1 G-allele  =  major frequency allele.

2 Fisher's exact test.

3 CD4+ T cell proliferation measured after 10 days incubation.

4 Cytokines measured in cell cultures supernatants after seven days stimulation with MBP.

5 Cytokine measured in cell cultures supernatants after one day of stimulation with MBP.

### Statistics

Prism 4 (GraphPad, San Diego, CA, USA) was employed for statistical analysis. The Mann-Whitney U-test was used for comparison of unpaired data, and the Kruskall-Wallis test was used for testing equality of medians among groups. Spearman's rank correlation coefficient was used to determine whether two sets of data were associated. The Fisher's exact test was used to calculate the distribution of dichotomic data (present versus not present). P-values <0.05 were considered significant.

## Results

### NOD2 polymorphisms

We examined 29 MS patients for three most common polymorphisms in the *NOD2* gene: rs2066842 (Pro268Ser), rs5743277 (Arg703Cys), and rs5743291 (Val955Ile). Proline 268 is situated in the NACHT domain, whereas arginine 703 and valine 955 are situated in the LRR domain. No carriers of the minor allele of the rs5743277 polymorphism were found among the included patients (Table 1). Fourteen patients were heterozygous carriers (C/T) of the rs2066842 polymorphism and two were homozygous carriers of the T-allele. Seven patients were heterozygous (G/A) for the A-allele of the rs5743291 polymorphism, while none were homozygous.

### Influence of rs5743291 and rs2066842 polymorphisms on MBP-induced cytokine responses

We examined whether polymorphisms in *NOD2* influenced MBP-induced cytokine responses by MNCs isolated from 29 MS patients. After 24 hours of incubation with antigen, a significant production of TNF-alpha occurred in all MNC cultures tested, IL-4 production was observed in cultures from nine patients (Table 2), IFN-gamma production was observed in 24 (not shown), whereas cultures from only three patients produced IL-2 (not shown), and none produced IL-5 or IL-17.

MBP-induced IL-10 production was found in cultures from all patients but one (Table 2).

**Figure 1:**
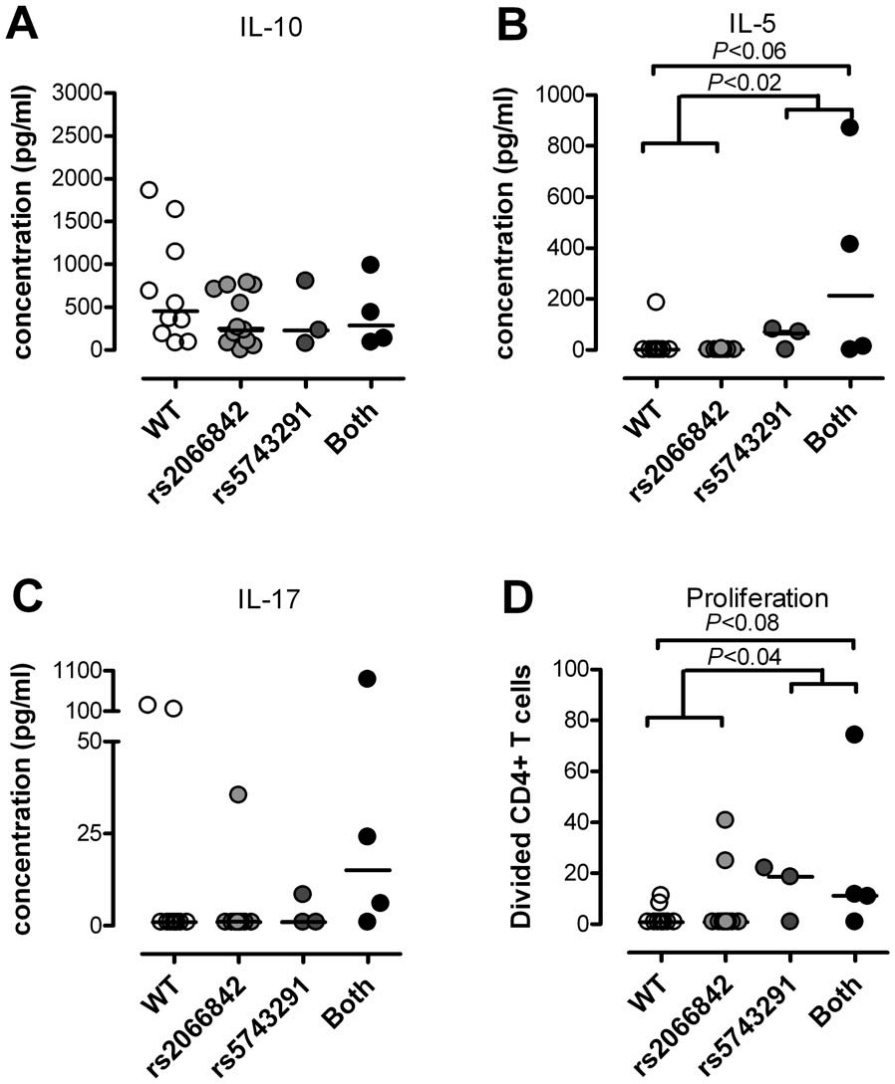
Influence of NOD2-polymorphisms on MBP-induced Th cell responses. CFSE-labeled mononuclear cells from twenty-nine patients were incubated for 10 days in medium containing (30% v/v) autologous serum and 30 µg/ml human MBP. (A) The contents of IL-10 in the culture supernatants were assessed after one day of incubation, and the contents of (B) IL-5 and (C) IL-17 were quantified after seven days. After 10 days of incubation, division of CD4+ T cells (D) was tracked by flow cytometry as cells having undergone more than one division. The data were grouped according to the patients' NOD2-polymorphisms: Nine were homozygous wildtypes for both rs2066842 and rs5743291 (WT; open circles), 13 carried the minor allele of rs2066842 only (light shaded circles), three carried the minor allele of rs5743291 (dark shaded circles), and four carried the minor allele of both polymorphisms (closed circles). Net values, after subtractions of background activities, are shown. Horizontal lines show the medians, and P-values were calculated using the Mann-Whitney U-test.

As shown in Figure 1A the IL-10 production was non-significantly reduced in patients carrying the minor allele of rs5743291 alone, rs2066842 alone or both (by 49%, 46% and 37%, respectively).

After seven days of stimulation with MBP, production of IL-5 had taken place in seven of 29 patient cultures (Table 2 and Fig. 1B), IFN-gamma in 14, IL-2 in eight, IL-17 in seven (Table 2), and IL-4 in four cultures (not shown).

As shown in Figure 1B, the MBP-induced production of IL-5 was significantly associated with the *NOD2* genotype (Kruskall-Wallis test: p<0.009). Grouped together, MNC cultures derived from carriers of minor alleles of the rs5743291 polymorphism displayed significantly higher contents of IL-5 (median 70 pg/ml) after stimulation with MBP than cultures from patients homozygous for the G-allele, in which the IL-5 content was, on median, undetectable (p<0.02; Fig. 1B). Accordingly, carriers of the G>A substitution in rs5743291 more frequently responded to MBP with IL-5 production than (G/G) homozygous patients (p<0.004; Table 2).

The MBP-elicited production of IL-17 was also associated with the *NOD2* genotypes (Fig. 1C), the median content of IL-17 in MNC cultures derived from patients carrying the minor allele of both polymorphisms being 15 pg/ml and undetectable in patients carrying one or no minor alleles of rs5743291 (Kruskall-Wallis test: p<0.09). IL-17 responses to MBP were thus observed in MNC cultures from four out of the seven patients carrying the minor A-allele of rs5743291 versus three out of 19 cultures from patients who were homozygous for the major allele (p<0.04; Table 2).

Carriers of the minor allele of rs2066842 alone did not show increased IL-5 responses or IL-17 responses.

We observed no association between either of the two polymorphisms and the production of IFN-gamma, IL-2, or IL-4 (Table 2; not shown for rs2066842).

### Influence of rs5743291 and rs2066842 polymorphisms on MBP-induced CD4+ T cell proliferation

**Figure 2:**
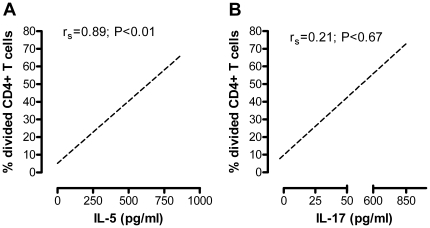
Correlation between MBP-induced Th-cell proliferation and production of IL-17 and IL-5. The MBP-induced IL-5 responses (A) and IL-17 responses (B) were compared with the CD4+ T cell proliferation of seven multiple sclerosis patients who were heterozygous for the minor A-allele of rs5743291. Closed circles (n = 4) represent patients who also carried the minor T-allele of the rs2066842 polymorphism. Spearman's rank correlation coefficient was used to determine association between data sets.

After 10 days of incubation with MBP, the proliferation of CD4+ T cells was assessed in the MS patient-derived cultures. As shown in Table 2, heterozygosity (G/A) in rs5743291 was associated with more frequent CD4+ T cell proliferative responses to MBP (p<0.02). Thus, five out of nine patients showing MBP-induced CD4+ T cell proliferation were heterozygous for this polymorphism, and three of them also carried the minor allele of rs2066842 (Fig. 1D). The five patients with CD4+ T cell proliferation were identical to those responding to MBP with IL-5 production (Fig. 2A), but only three of them responded with IL-17 production (Fig. 2B). The remaining four responders were equally distributed between patients carrying the minor allele of rs2066842 alone and patients homozygous for the major allele.

As reported previously, 14 of the patients were followed for six months after initiation of IFN-beta therapy [25]. During this period, an additional four patients exhibited MBP-induced CD4+ T cell proliferation, including the two heterozygous patients without pretreatment response. Thus all seven heterozygous (G/A) patients responded to MBP with CD4+ T cell proliferation at one or more time points within the observation period versus only six patients who were homozygous for the major allele (p<0.002; not shown).

### Circulating cytokine levels

To test whether the exaggerated MBP-induced cytokine responses in patients carrying the minor allele of rs5743291 were reflected by increased levels of circulating cytokines *in vivo*, we measured IL-2, IL-4, IL-5, IL-10, IL-17, TNF-alpha, and IFN-gamma in sera from 27 of the patients included in the study; seven who carried the minor allele of the rs5743291 polymorphism, and twenty who were homozygous for the major allele. IFN-gamma (5–12 pg/ml) was found in three sera; from a patient who were heterozygous for the minor alleles of both rs5743291 and rs2066842 and from two patients who carried neither of the minor alleles (NS). A fourth patient, heterozygous for both minor alleles, had detectable IL-10 levels (10 pg/ml; data not shown). No other circulating cytokines were detected (data not shown). None of the patients displayed spontaneous CD4+ T cell proliferation.

## Discussion

Mutations in *NOD2* have been associated with chronic inflammatory disorders such as Crohn's disease and Blau syndrome. Following our previous findings that a MNCs from a subgroup of MS patients respond to challenge with MBP ex vivo with CD4+ T-cell proliferation and production of IL-17, IFN-γ, IL-5 and IL-4 [18], we have here examined whether these responses are associated with the three most common *NOD2* mutations. The influence of rs5743277 could not be investigated, since all patients examined were homozygous for the major allele.

The rs2066842 polymorphism did not significantly influence the cellular responses to MBP, while patients carrying the minor allele of rs5743291, resulting in a substitution of Valine955 with isoleucine in the LRR domain, displayed excessive T cell responses, as illustrated by enhanced CD4+ T cell proliferation.

We were unable to demonstrate associations between the polymorphisms and MBP-induced Th1-responses but did observe enhanced IL-5 and, notably, IL-17 responses in patients who were heterozygous for the G>A substitution in rs5743291. Concomitantly, these patients displayed a non-significant reduction (by 37–49%) of the IL-10 production, in keeping with previous findings that mutations in the LRR domain of *NOD2* are associated with reduced expression of this cytokine [10]. While IL-10 is known to play a protective role in MS [26], we have previously observed that the level of MBP-induced IL-5 correlated positively with disease activity [18], and that T cells from secondary progressive MS patients have a higher expression of IL-5 than those of RRMS patients [27]. On the other hand, IL-5 is upregulated during glatiramer acetate therapy and does not appear to increase during relapses like another Th2 cytokine, IL-13 [28], [29]. It is possible that Th2 cells play different roles at different stages of MS.

The role of Th17 cell in the pathogenesis of MS has been intensively investigated during recent years. For example, inflammation in the brain parenchyma apparently occurs when Th17 cells outnumber Th1 cells, resulting in elevated IL-17 expression, and IL-17 production correlates with disease activity [18], [30], [31]. Thus, the exaggerated IL-17 response to MBP in four out of seven patients carrying the minor allele of rs5743291 is likely to have detrimental consequences.

The exaggerated production of IL-5 and IL-17 in cultures derived from MS patients heterozygous for the G>A substitution in rs5743291 were not reflected by detectable levels of these cytokines in the circulation.

It was not possible to include a group of healthy controls to examine the relationship between *NOD2* polymorphism and MBP-induced CD4+ T-cell proliferation or production of IL-17, IFN-γ, IL-5 and IL-4, since MBP induces these responses in patient-derived cultures exclusively [18].

To our knowledge, the present study demonstrates for the first time that T cell proliferative responses and IL-5/IL-17 responses to a myelin self-antigen are linked with NOD2 polymorphism. It is known that mutations in LRR of the encoded protein NOD2 are associated with reduced production of IL-10, which plays a protective (antiinflammatory) role in MS [10], [26].

Our study was hampered by the low number of MS patients included and the lack of patients homozygous for the minor alleles of the polymorphisms examined (none for rs5743277 and rs5743291, and only two for rs2066842). Therefore, the results can only be regarded as hypothesis-generating, and studies of larger cohorts or more homozygous patients are needed to certify associations between the polymorphisms and Th1- and IL-10 responses to MBP or other MS-associated self-antigens. Moreover, the mechanisms behind the apparent effect of NOD2 polymorphism and IL-5/IL-17 production need to be elucidated.

In summary, our data indicate that MNCs derived from MS patients, who are heterozygous for a G>A substitution in exon 9 of the NOD2 gene, display enhanced CD4+ T cell proliferation and production of IL-5 and IL-17 in response to challenge with the myelin self-antigen MBP. Studies in larger cohorts of patient are needed to confirm this hypothesis. While the enhanced IL-17 production may have pathophysiological implications, the role of IL-5 in MS needs further investigation.
